# The BPPV-SQ: Development and Clinical Evaluation of a Brief Screening Questionnaire for Benign Paroxysmal Positional Vertigo

**DOI:** 10.3390/audiolres16020058

**Published:** 2026-04-11

**Authors:** Giacinto Asprella-Libonati, Fernanda Asprella-Libonati, Marco Familiari, Vito Rizzi, Camilla Gallipoli, Margherita Laguardia, Giuseppe Gagliardi, Anna Guida, Giuseppe Lapacciana, Luca Colella, Giada Cavallaro

**Affiliations:** 1 Otolaryngology Unit, Madonna Delle Grazie Hospital of Matera, 75100 Matera, Italy; vito.rizzi@asmbasilicata.it (V.R.); camilla.gallipoli@asmbasilicata.it (C.G.); margherita.laguardia@asmbasilicata.it (M.L.); giuseppe.gagliardi@asmbasilicata.it (G.G.); annac.guida@asmbasilicata.it (A.G.); giuseppe.lapacciana@asmbasilicata.it (G.L.); giada.cavallaro@asmbasilicata.it (G.C.); 2 Otolaryngology Unit, Engles Profili Hospital, AST Ancona, 60044 Fabriano, Italy; fernandasprella@gmail.com; 3 UOSD Vestibologia e Otorinolaringoiatria Presidio Ospedaliero Giovanni Paolo II, 75025 Policoro, Italy; marco.familiari@asmbasilicata.it (M.F.); luca.colella@asmbasilicata.it (L.C.)

**Keywords:** questionnaire, BPPV, screening

## Abstract

**Background**: Benign paroxysmal positional vertigo (BPPV) is the most common cause of peripheral vertigo and is diagnosed clinically, yet many patients initially present in primary care. Early identification may optimize referral and management. **Objective**: To perform a pilot Phase 1 validation of the Benign Paroxysmal Positional Vertigo Screening Questionnaire (BPPV-SQ), a brief screening questionnaire designed for future use in general practice (primary care settings where patients are initially evaluated by general practitioners), assessing its ability to identify BPPV, suggest canal involvement, and support progression to Phase 2 validation. **Methods**: In this prospective observational study, 108 patients with positional vertigo and no neurological signs were evaluated in a specialist setting. The 7-item dichotomous questionnaire (score 0–3 for diagnostic core) was administered prior to bedside examination, which served as the reference standard. **Results**: Higher questionnaire scores were associated with an increased probability of confirmed BPPV. Among patients with the maximum score of 3, BPPV was confirmed in 78.3% of cases, with a lateralization concordance of 69.4% between questionnaire responses and specialist diagnosis. In contrast, lower scores (0–1) were associated with a markedly lower rate of confirmed BPPV (14.3%). **Conclusions**: In this pilot Phase 1 validation, the BPPV-SQ demonstrated score-dependent diagnostic reliability and acceptable lateralization agreement in high-score patients, supporting progression to Phase 2 validation in primary care.

## 1. Introduction

Benign paroxysmal positional vertigo (BPPV) is the most frequent cause of peripheral vestibular vertigo and is typically attributed to canalolithiasis or cupulolithiasis, conditions in which displaced otoconia from the utricle migrate into a semicircular canal or adhere to the cupula [1]. The resulting abnormal endolymphatic dynamics generate brief episodes of positional vertigo associated with characteristic nystagmus patterns [2]. Diagnosis is primarily clinical and relies on a detailed history combined with the identification of positional nystagmus elicited by specific maneuvers. Accurate localization of the affected ear and semicircular canal is essential for appropriate therapeutic repositioning [1]. In routine clinical practice, a substantial proportion of patients with dizziness initially consult general practitioners (GPs) [3]. The definitive diagnosis of BPPV is typically established through bedside positional testing, including maneuvers such as the Dix–Hallpike test and the supine roll test, which allow clinicians to identify characteristic positional nystagmus and determine the affected semicircular canal. However, these maneuvers are not always performed during the initial evaluation in primary care. In this context, time constraints and limited access to vestibular diagnostic equipment may make it difficult to perform a complete vestibular assessment during the initial consultation. A simple screening instrument capable of identifying patients with a high probability of BPPV could therefore assist clinicians in prioritizing referral for specialist evaluation and treatment. The present study represents a Phase I validation, defined as an initial pilot evaluation of the questionnaire in a specialist-controlled clinical setting.

The primary objective of this pilot Phase 1 validation study was to evaluate the diagnostic performance of a brief screening questionnaire (BPPV-SQ) designed for future use in primary care. Specifically, the study aimed to determine whether the questionnaire could identify patients with clinical features consistent with BPPV, assess its ability to suggest the involved semicircular canal and side lateralization, and evaluate whether the instrument demonstrates sufficient diagnostic performance to justify progression to a Phase 2 validation study in general practice. Particular attention was given to potential confounding factors that may influence the diagnostic evaluation of vertigo, including the presence of logical symptoms, a history of central nervous system disorders, and atypical vertigo characteristics. Patients presenting with such features were excluded in order to minimize diagnostic uncertainty and ensure that the questionnaire was tested primarily in subjects with suspected peripheral positional vertigo.

## 2. Materials and Methods

### 2.1. Study Design and Rationale (Pilot Phase 1 Validation Study)

This prospective observational study represents the pilot Phase 1 validation study of the BPPV-SQ. The study was conducted at the Otoneurology Units of Matera and Policoro (Basilicata, Italy). The questionnaire was validated in a specialist otoneurological clinical setting, where patients were evaluated by physicians experienced in vestibular disorders and where standardized diagnostic maneuvers and appropriate examination equipment were available.

Patients were consecutively enrolled if they presented with vertigo suggestive of positional origin. All patients were referred from the Emergency Department to the Otoneurology Units of Matera and Policoro for specialist evaluation.

Only patients without current neurological signs (e.g., persistent non-positional vertigo, neurological deficits, severe headache, diplopia, dysarthria, limb weakness, or atypical nystagmus patterns not compatible with peripheral positional vertigo) or a previous history suggestive of central nervous system pathology were included.

Patients with suspected central vertigo identified during the initial clinical assessment were excluded.

Adult patients were included, with no strict upper age limit, reflecting real-world clinical referral patterns.

The rationale for this pilot Phase 1 validation study was to test the questionnaire in a controlled clinical environment and to evaluate its diagnostic agreement with specialist bedside assessment, which served as the reference standard. In addition, the study aimed to estimate whether a sufficiently high proportion of positive correspondence—preliminarily considered to be ≥60–70% in the high-score categories—could justify progression to a Phase 2 validation study in general practice.

### 2.2. Study Population

A total of 108 consecutive patients presenting with vestibular complaints were enrolled (56 women, 52 men; mean age 50.2 years, range 29–87 years).

Although recent onset (≤15 days) was considered ideal for future application, this pilot Phase 1 validation study included consecutive eligible patients meeting the inclusion criteria.

### 2.3. Questionnaire Structure

The BPPV-SQ (Table 1) is a brief screening questionnaire composed of seven dichotomous items (Yes/No) designed to identify patients with clinical features suggestive of benign paroxysmal positional vertigo (BPPV).

The questionnaire is structured into three functional domains.

- Diagnostic Core (Items 1–3).

The first three items represent the diagnostic core of the questionnaire and investigate the most characteristic symptoms of BPPV:

Vertigo triggered by head movements.

Rotatory sensation.

Brief duration of vertigo (≤3 min).

Each positive answer is assigned 1 point, generating a diagnostic score ranging from 0 to 3.

The diagnostic score is interpreted as follows:

0—No BPPV.

1—Possible BPPV.

2—Probable BPPV.

3—Definite BPPV.

These items represent the primary screening component of the questionnaire and were the main variables used to evaluate diagnostic performance in this pilot study.

- Canal Suggestion (Items 4–5)

Items 4 and 5 explore the type of positional movement that triggers vertigo, with the aim of suggesting the potentially involved semicircular canal.

Item 4 investigates movements typically associated with posterior semicircular canal BPPV, such as bending forward, looking upward, getting out of bed, or lying down.

Item 5 explores vertigo triggered by turning the head or body to one side in bed, a pattern more frequently associated with lateral semicircular canal BPPV.

These questions were included to generate a preliminary clinical hypothesis regarding canal involvement, although this aspect was considered exploratory in the present pilot study.

- Lateralization (Items 6–7)

Items 6 and 7 investigate the possible side predominance of symptoms.

Item 6 asks whether vertigo is worse when turning onto a specific side.

Item 7 explores the patient’s preferred sleeping side.

These items were used to evaluate lateralization agreement between the questionnaire responses and the side of involvement identified during specialist clinical examination. The questionnaire was originally developed in Italian and subsequently translated into English for publication purposes. All patients included in the present study completed the Italian version. The BPPV-SQ was specifically developed for the purposes of the present study, based on the typical clinical history of BPPV patients and designed to remain short and easy to administer in primary care.

### 2.4. Reference Standard

The final diagnosis of BPPV was established through a specialist bedside vestibular examination performed by experienced otoneurology clinicians using Frenzel goggles.

Standard positional maneuvers were performed, including:

The Dix–Hallpike maneuver for the diagnosis of posterior semicircular canal BPPV.

The Supine Head Roll Test (Pagnini–McClure maneuver) for the identification of lateral semicircular canal BPPV.

A diagnosis of BPPV was confirmed when typical positional vertigo associated with characteristic positional nystagmus was observed during these maneuvers.

The results of this clinical examination were used as the reference standard for evaluating the diagnostic performance of the BPPV-SQ.

### 2.5. Statistical Analysis

Descriptive statistics were calculated as absolute numbers and percentages.

Diagnostic performance was evaluated by comparing questionnaire results (particularly score = 3 and score ≥ 2) with the final clinical diagnosis. Sensitivity, specificity, positive predictive value (PPV), and negative predictive value (NPV) were estimated for selected score thresholds (score = 3 vs. <3; score ≥ 2 vs. <2). Agreement between questionnaire-based lateralization and specialist clinical findings was assessed descriptively as percentage concordance. Sensitivity, specificity, PPV, and NPV were estimated for selected score thresholds by comparing questionnaire results with the final clinical diagnosis.

### 2.6. Ethical Considerations

All patients provided written informed consent for the collection and use of their clinical data for research purposes. The study was conducted in accordance with the ethical principles of the Declaration of Helsinki.

## 3. Results

Among the 108 patients:

Score 0:14 patients (12.96%).

BPPV confirmed in 2 cases (14.29%).

Score 1: 14 patients (12.96%).

BPPV confirmed in 2 cases (14.29%).

Score 2: 34 patients (31.48%).

BPPV confirmed in 8 cases (23.53%).

Lateralization concordance: 3/8 cases (37.50%).

Score 3: 46 patients (42.59%).

BPPV confirmed in 36 cases (78.26%).

Lateralization concordance: 25/36 cases (69.44%).

Overall, higher questionnaire scores were associated with an increased probability of confirmed BPPV. When considering a threshold of score = 3 as positive, the questionnaire demonstrated a positive predictive value of approximately 78%, with a progressive increase in diagnostic probability across score categories. Lower scores (0–1) were associated with a low probability of confirmed BPPV, suggesting potential utility in excluding clinically evident cases in selected contexts.

## 4. Discussion

Patients with dizziness frequently present first to primary care services. In this context, the GPs play a pivotal role in distinguishing peripheral vestibular disorders from alternative etiologies, including central causes that may require urgent investigation [4]. Several validated questionnaires have been proposed to aid in the evaluation of dizzy patients [5,6,7,8]. Recently, Yan et Al. Developed a questionnaire for self-diagnosis of BPPV, which can be used for self-application of appropriate CRP by determining the affected ear and subtype of BPPV when it occurs and recurs [9]. To the best of our knowledge, the literature has not yet reported a simple and user-friendly questionnaire specifically designed for use in GP settings, aimed at patients presenting with symptoms suggestive of positional vertigo. This pilot Phase 1 validation study represents the first structured validation step of the BPPV-SQ in a specialist-controlled setting prior to its implementation in primary care. The objective of this pilot Phase 1 validation study was not to provide definitive estimates of diagnostic accuracy but to assess feasibility, internal coherence, and agreement with specialist diagnosis as a reference standard.

### 4.1. Feasibility

Feasibility was indirectly assessed through:

Successful administration of the questionnaire to all 108 enrolled patients.

Short administration time and the possibility of self-administration or minimal assistance.

Absence of missing responses or difficulties in questionnaire completion.

These aspects support the potential suitability of the BPPV-SQ for use in primary care settings.

### 4.2. Internal Coherence

Internal coherence was explored qualitatively through the observed score-dependent diagnostic trend, with increasing probability of confirmed BPPV as the diagnostic score increased from 0 to 3. This progressive gradient supports the conceptual structure of the diagnostic core items.

The findings indicate:

- A progressive increase in the proportion of confirmed BPPV cases was observed across increasing questionnaire score categories;
- Good diagnostic reliability in patients with the maximum score (3);
- Lateralization concordance between questionnaire responses and specialist diagnosis reached approximately 69% among patients with the highest diagnostic score, indicating a moderate to good level of agreement.

These results suggest that the BPPV-SQ may have practical value as a triage tool, particularly when high scores are obtained, and support progression to a Phase 2 validation study in general practice.

However, the study has several limitations:

- Relatively small sample size;
- Single-region specialist setting;
- Absence of immediate validation in primary care;
- Inclusion of patients not strictly limited to very recent onset in all cases.

Further studies are required to evaluate sensitivity, specificity, and predictive values in broader and less selected populations.

## 5. Conclusions

In conclusion, the BPPV-SQ is a brief and easily administrable screening questionnaire designed for potential use in primary care settings. In this pilot Phase 1 validation study, the instrument demonstrated a clear score-dependent increase in the probability of confirmed BPPV, good diagnostic reliability in patients with the highest scores, and acceptable agreement with specialist-determined lateralization. These preliminary findings support progression to a Phase 2 validation study in which the questionnaire will be directly tested in general practice settings. These findings support progression to a Phase 2 validation study in general practice, in which the questionnaire will be directly administered by general practitioners to patients presenting with recent-onset positional vertigo, in order to evaluate its real-world performance, feasibility, and impact on referral pathways. Larger, multicenter studies will subsequently be required to better define its sensitivity, specificity, and predictive values in real-world primary care settings.

## Figures and Tables

**Table 1:** Italian and English versions of the questionnaire.

QUESTIONARIO DI SCREENING PER PAZIENTI CON SOSPETTA VPPB BPPV-SQ
1D. Italiano: La vertigine è scatenata dai movimenti della testa, come quando ci si sdraia a letto, quando ci si alza dal letto, piegandosi in avanti con la testa, sollevando la testa verso l’alto o girandosi nel letto su un lato? English: Is your vertigo triggered by head movements, such as when lying down in bed, getting out of bed, bending forward, looking upward, or turning over in bed onto one side? a. Sì/Yes b. No/No
2D. Italiano: Quando hai le vertigini, senti come se tutto ti girasse intorno? English: When you experience vertigo, does it feel as though everything is spinning around you? a. Sì/Yes b. No/No
3D. Italiano: La vertigine è breve e dura al massimo tre minuti? English: Is the vertigo brief, lasting no more than three minutes? a. Sì/Yes b. No/No
4C. Italiano: La vertigine è scatenata dal movimento della testa quando ci si piega in avanti, si solleva la testa verso l’alto, ci si alza dal letto o ci si sdraia a letto? English: Is the vertigo triggered by head movement when bending forward, looking upward, getting out of bed, or lying down in bed? a. Sì/Yes b. No/No
5C. Italiano: La vertigine è scatenata dal movimento della testa girandosi su un lato nel letto o girando la testa da un lato? English: Is the vertigo triggered by turning over onto one side in bed or by turning your head to one side? a. Sì/Yes b. No/No
6L. Italiano: La vertigine è peggiore quando ci si gira su un lato specifico? English: Is the vertigo worse when turning onto one particular side? a. Sì—lato destro/lato sinistro Yes—right side/left side b. No/No
7L. Italiano: Preferisci dormire su un lato? English: Do you prefer to sleep on one side? a. Sì—lato destro/lato sinistro Yes—right side/left side b. No/No
Punteggio/Scoring Sì = 1 No = 0 Yes = 1 No = 0 Questions 1–2–3 → D (Diagnosi di VPPB/Diagnosis of BPPV) Question 4 → Canale posteriore/Posterior canal Question 5 → C (Canale laterale/Lateral canal) Questions 6–7 → L (Lato/Side involved) Total Score (Diagnostic Questions D 1–2–3) 0 = Non VPPB/No BPPV 1 = VPPB possibile/Possible BPPV 2 = VPPB probabile/Probable BPPV 3 = VPPB certa/Definite BPPV

## Data Availability

The original contributions presented in this study are included in the article. Further inquiries can be directed to the corresponding author.
