# Correction: Asprella-Libonati et al. The BPPV-SQ: Development and Clinical Evaluation of a Brief Screening Questionnaire for Benign Paroxysmal Positional Vertigo. *Audiol. Res.* 2026, *16*, 58

**DOI:** 10.3390/audiolres16060165

**Published:** 2026-10-30

**Authors:** Giacinto Asprella-Libonati, Fernanda Asprella-Libonati, Marco Familiari, Vito Rizzi, Camilla Gallipoli, Margherita Laguardia, Giuseppe Gagliardi, Anna Guida, Giuseppe Lapacciana, Luca Colella, Giada Cavallaro

**Affiliations:** 1 Otolaryngology Unit, Madonna Delle Grazie Hospital of Matera, 75100 Matera, Italy; vito.rizzi@asmbasilicata.it (V.R.); camilla.gallipoli@asmbasilicata.it (C.G.); margherita.laguardia@asmbasilicata.it (M.L.); giuseppe.gagliardi@asmbasilicata.it (G.G.); annac.guida@asmbasilicata.it (A.G.); giuseppe.lapacciana@asmbasilicata.it (G.L.); giada.cavallaro@asmbasilicata.it (G.C.); 2 Otolaryngology Unit, Engles Profili Hospital, AST Ancona, 60044 Fabriano, Italy; fernandasprella@gmail.com; 3 UOSD Vestibologia e Otorinolaringoiatria Presidio Ospedaliero Giovanni Paolo II, 75025 Policoro, Italy; marco.familiari@asmbasilicata.it (M.F.); luca.colella@asmbasilicata.it (L.C.)

There was an error in the original publication [1] due to an inadvertent transcription error in the number of patients assigned a diagnostic BPPV-SQ score of 3. The published Results reported 49 patients in this category, whereas verification against the original paper-based study database confirmed 46 patients. The total study population was correctly reported as 108 patients, and the number of clinically confirmed BPPV cases in the score-3 group remains 36.

Corrections have been made to Section 3 (Results), in the paragraph reporting the distribution of diagnostic scores, and to the Abstract (Results) to reflect the corrected proportion of confirmed BPPV cases among patients with a score of 3:

Score 0: 14 patients (12.96%). BPPV confirmed in 2 cases (14.29%). Score 1: 14 patients (12.96%). BPPV confirmed in 2 cases (14.29%). Score 2: 34 patients (31.48%). BPPV confirmed in 8 cases (23.53%). Lateralization concordance: 3/8 cases (37.50%). Score 3: 46 patients (42.59%). BPPV confirmed in 36 cases (78.26%). Lateralization concordance: 25/36 cases (69.44%). When considering a threshold of score = 3 as positive, the questionnaire demonstrated a positive predictive value of approximately 78%, with a progressive increase in diagnostic probability across score categories. Accordingly, the Abstract should state: “Among patients with the maximum score of 3, BPPV was confirmed in 78.3% of cases, with a lateralization concordance of 69.4% between questionnaire responses and specialist diagnosis.”

The authors state that the scientific conclusions are unaffected. This correction was approved by the Academic Editor. The original publication has also been updated.

## References

[1] Asprella-Libonati G., Asprella-Libonati F., Familiari M., Rizzi V., Gallipoli C., Laguardia M., Gagliardi G., Guida A., Lapacciana G., Colella L. (2026). The BPPV-SQ: Development and Clinical Evaluation of a Brief Screening Questionnaire for Benign Paroxysmal Positional Vertigo. Audiol. Res..

